# Preparation and Modification of Sucrose-Based Non-Isocyanate Polyurethane Adhesives for Plywood Bonding

**DOI:** 10.3390/molecules30071541

**Published:** 2025-03-30

**Authors:** Hongyi Zhong, Qianyu Zhang, Hong Lei, Xiaojian Zhou, Jun Zhang, Guanben Du, Antonio Pizzi, Xuedong Xi

**Affiliations:** 1 Yunnan Key Laboratory of Wood Adhesives and Glued Products, College of Material Science and Chemistry Engineering, Southwest Forestry University, Kunming 650224, China; 13547938329@163.com (H.Z.); zqy20160922@163.com (Q.Z.); xiaojian.zhou@swfu.edu.cn (X.Z.); zj8101274@163.com (J.Z.); guanben.du@swfu.edu.cn (G.D.); 2 School of Chemistry and Material Engineering, Zhejiang A&F University, Hangzhou 311300, China; leihong@zafu.edu.cn; 3 LERMAB, University of Lorraine, 88000 Epinal, France

**Keywords:** biomass adhesive, NIPU, sucrose, bonding strength

## Abstract

The production of non-isocyanate polyurethane (NIPU) resins using recyclable biomass materials and no isocyanates as a substitute for traditional polyurethane (PU) materials has become a research focus in the polyurethane industry. The development of such NIPU resins for application as wood adhesives has also emerged as an interesting new research topic. In this study, sucrose was used to react with dimethyl carbonate, and then polymerized with an amine to prepare sucrose-based non-isocyanate polyurethane (SNIPU) adhesives and evaluate their suitability for use in plywood. Four amines, namely polyethylene amine (PEI) of molecular weight (MW) 10,000, PEI of MW 1800, diethylenetriamine, and hexanediamine were tested in the preparation of SNIPU adhesives to determine a more suitable amine showing optimal adhesion performance. The effect of the amount of the amine added on adhesive properties was further investigated. The results showed that the SNIPU adhesive prepared with PEI-10000 as amine presents a good bonding performance. The SNIPU prepared with a PEI-10000 content of 45% (*w/w* on sucrose) presented the highest bonding strength. The dry strength, 24 h cold water (23 °C) wet strength, and 3 h hot water (63 °C and 93 °C) wet strengths of its bonded plywood were 1.26 MPa, 0.90 MPa, 0.84 MPa, and 0.80 MPa, respectively. Furthermore, the addition of 13% (*w/w* on SNIPU adhesive) of ethylene glycol diglycidyl ether (EGDE) as a modifier showed a significant decrease of 20 °C of the curing temperature of the SNIPU adhesive.

## 1. Introduction

Polyurethanes (PU) are high-performance polymers formed by polycondensation of polyols and isocyanates. Due to their excellent mechanical properties and high plasticity, they are widely used in the wood-based panels industry, including coatings and adhesives [1,2]. However, isocyanates, a raw material used in PU, are highly toxic and volatile. To avoid their potential dangers, research into environmentally friendly and high-performance polyurethanes (PU) has become a significant trend. Consequently, non-isocyanate polyurethane (NIPU) wood adhesives that do not use isocyanates as raw materials have become a focal point of research. Currently, the reaction of cyclic carbonates with aliphatic primary amines has become one of the mainstream methods for synthesizing NIPUs due to its safety and environmental benefits. Furthermore, in the context of green and sustainable development, utilizing natural renewable resources through the reaction of cyclic carbonates and aliphatic primary amines to prepare sustainable NIPU adhesives holds a significant research interest [3,4,5,6,7].

Natural biomass materials are widely regarded as good substitutes for non-renewable and toxic substances due to their broad availability, low cost, and lack of toxicity [8,9,10]. There are reports of using natural tannin as a raw material to prepare a tannin-based non-isocyanate adhesive [11]. However, tannin raw extract is a macromolecular polyphenol containing pectin carbohydrates, which normally results in an excessively high viscosity of the tannin-based non-isocyanate adhesive [12]. Additionally, a lignin-based non-isocyanate polyurethane resin prepolymer with both branched and straight chain architectures was developed utilizing lignin as raw material [13]. Lignin presents fewer reaction sites and lower reactivity due to the many substituents on its aromatic rings of lignin. This results in a poor adhesive performance when used in the production of particleboard. Due to these disadvantages, its use for wood gluing when alone is not ideal. Thus, to find a chemical biomass feedstock with more reactive sites is a worthwhile endeavor.

Sucrose is a smaller size carbohydrate with eight hydroxyl groups that can directly serve as reaction sites for adhesive preparation. Consequently, sucrose is gradually being used as a biomass raw material in the production of wood adhesives [14,15,16]. Additionally, sucrose is a commercially and widely available raw material also found in the leaves, flowers, stems, seeds, and fruits of plants, with particularly high concentrations in sugarcane, sugar beets, and maple sap [17,18]. Due to its abundant availability and low cost, sucrose is extensively used in the beverage, food, and chemical industries [19,20,21,22]. In previous work, good wood bonding sucrose-based non-isocyanate polyurethanes were developed by reacting sucrose with dimethyl carbonate and hexamethylenediamine. However, hexamethylenediamine has a relatively low molecular weight with only two amine groups resulting in a relatively low level of condensation resulting in a relatively higher curing temperature and longer curing time than is useful for wood panel adhesives. Moreover, unreacted hexamethylene diamine might volatilize during hot pressing, posing a hazard to the environment and human health [23]. Therefore, to address these issues, selecting a high-molecular-weight amine with a suitably higher degree of polymerization as a raw material for preparing sucrose-based NIPU adhesives is an interesting research direction.

Polyethyleneimine (PEI) is a water-soluble polymer comprising a large number of repeating structural units and a substantial number of amino groups [24]. The ultra-high branching of PEI is conducive to enhanced reactivity and higher levels of crosslinking [25]. PEI has polar (amino) and hydrophobic groups that enable crosslinking with a number of compounds. Because of this, PEI presents advantages in the preparation of adhesives and can thus also be employed in wood adhesives [26,27].

Thus, in the research work presented here, a novel sucrose-based NIPU adhesive was prepared from sucrose, dimethyl carbonate (DMC), and PEI-10000 as the primary raw materials. Unlike conventional polyurethane adhesives, this approach completely avoids the use of toxic isocyanates, making it more environmentally friendly and sustainable. The impact of incorporating diverse polyamine compounds and varying quantities of PEI-10000 on the adhesive’s functionality was systematically examined, and the synthesis procedure was refined to enhance its performance. Moreover, the addition of EGDE as an additional cross-linker has been strategically introduced, leading to further optimization of the bonded panels’ hot-pressing conditions. This study not only provides a sustainable alternative to traditional adhesives but also offers new insights into the molecular design and processing optimization of non-isocyanate polyurethanes, addressing critical challenges in bio-based adhesive technology.

## 2. Results and Discussions

### 2.1. Effect of Polyamine Type on SNIPU Adhesives

**Figure 1:**
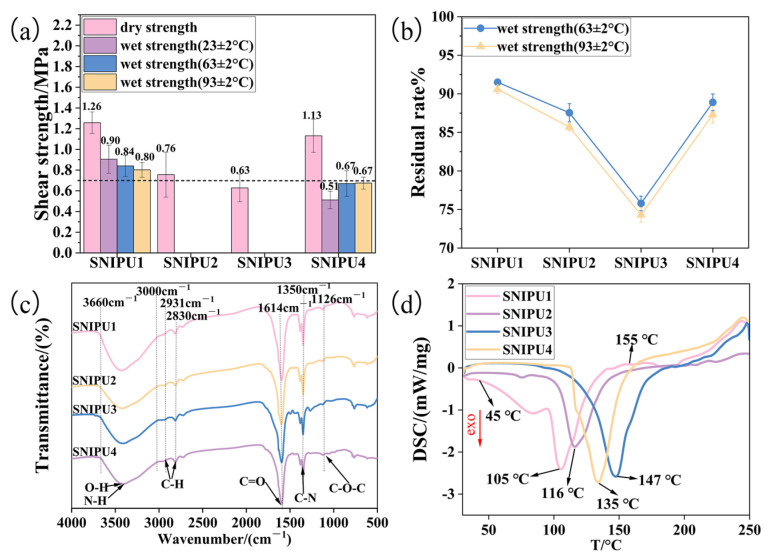
(**a**) Shear strength of SNIPU adhesives. (**b**) The water resistance of cured adhesivestest results of SNIPU adhesives. (**c**) FT-IR curves of SNIPU adhesives. (**d**) DSC curves of SNIPU adhesives.

Figure 1a shows the shear strength test results of SNIPU adhesives prepared with different polyamines. The plywood bonded with the SNIPU1 adhesive synthesized from PEI-10000 showed the best bonding performance. Its dry strength, 24 h cold water (23 °C) wet strength, and 3 h hot water (63 °C and 93 °C) wet strengths were 1.26 MPa, 0.90 MPa, 0.84 MPa, and 0.80 MPa, respectively, compliant with the national standard GB/T 9846-2015 [28] (dashed line in Figure 1a). However, the other three adhesives did not show a good bond strength, and in particular a poor wet shear strength. Water and moisture resistance are critical parameters that determine whether the adhesive can be used in practice as they affect plywood durability [29]. Figure 1b shows the water resistance of cured adhesives test results of SNIPU adhesives synthesized from different types of amines and indicates that the two PEIs present better water resistance of cured adhesives of lower-molecular-weight linear diamines and triamines. The SNIPU1 adhesive synthesized from PEI-10000 achieved a water resistance of cured adhesives of over 90%, demonstrating good water resistance of cured adhesives, which suggests that reactions with macromolecular amines increase the polymerization degree, thus enhancing the water resistance of cured adhesives. This also supports the test results shown in Figure 1a, indicating that it has superior water resistance of cured adhesives compared to adhesives synthesized from other amines.

Figure 1c shows the FTIR spectra characterizing the chemical structures of the different SNIPU adhesives. The absorption bands in the region 3000 cm^−1^–3660 cm^−1^ are generated by O-H and N-H stretching vibrations; the peaks 2830 cm^−1^ and 2931 cm^−1^ are generated by the stretching vibrations of C-H; the 1614 cm^−1^ peak is attributed to the absorption peak from the C=O stretching vibration. A distinct absorption peak at 1350 cm^−1^ is a C-N stretching vibration; the peak at 1126 cm^−1^ is the characteristic absorption peak of C-O-C in polyether groups [30,31]. The stretching vibration of C=O at the peak of 1614 cm^−1^ is indicative of the production of the carbamate structure. Under the combined influence of hydrogen bonds in amino and carbamate structures, the absorption peak shifts to a lower wavenumber. Additionally, hydrogen bonding facilitates the formation of intramolecular six-membered rings, resulting in a more stable structure with improved hydrolytic and chemical resistance [32,33,34]. Therefore, in Figure 1c, as the SNIPU1 adhesive presents a more marked 1614 cm^−1^ peak, this appears to indicate the formation of more carbamate structures. This is one reason why it shows a better bonding performance than the other SNIPU adhesives.

Figure 1d shows the DSC analysis results investigating the effect of the different polyamines on the thermal properties of SNIPU adhesives. It can be observed that the SNIPU1 adhesive’s curing peak temperature (105 °C) was significantly lower than that of the other three adhesives (116 °C, 135 °C, 147 °C). Moreover, its curing onset temperature (45 °C) and curing end temperature (155 °C) were lower, thus completing the adhesive curing earlier, implying that SNIPU1 cures more rapidly at a lower temperature than the others. This is a key factor in ensuring the rapid formation of strong bonds between wood surfaces. Furthermore, comparing the areas of the DSC curves for the four adhesives, the area for the SNIPU1 adhesive is significantly larger than the others, exhibiting a larger exothermic peak, indicating that its exothermal curing reaction is more intense and that its curing is more complete [35]. Thus, this means that under the same hot-press conditions, the curing and bonding performance of the SNIPU1 adhesive is superior to that of the other adhesives. This confirms the reasonable insight of selecting PEI-10000 as the polyamine in this work. Thus, PEI-10000 can be considered as the preferred polyamine for the preparation of SNIPU adhesives. An endothermic peak appears after 200 °C, primarily due to the evaporation of moisture in the adhesive and the decomposition of the adhesive.

### 2.2. Effect of PEI-10000 Dosage on Adhesives

**Figure 2:**
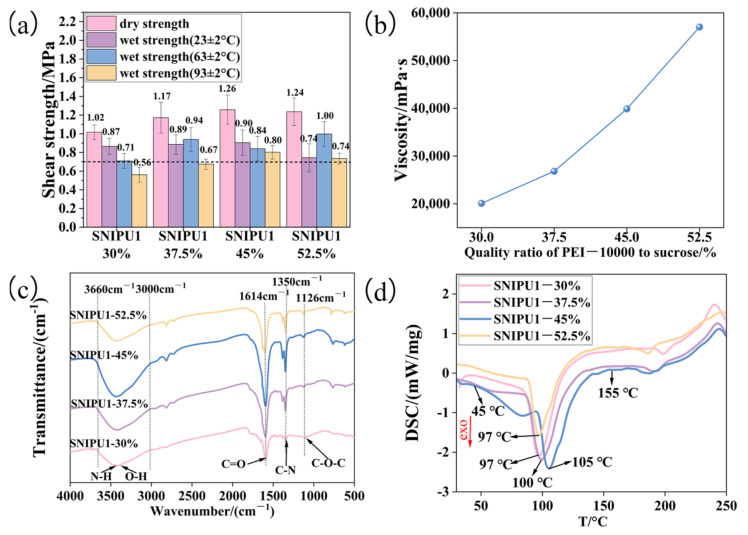
(**a**) Shear strength of SNIPU1 adhesives. (**b**) Viscosity of SNIPU1 adhesives. (**c**) FT-IR curves of SNIPU1 adhesives. (**d**) DSC curves of SNIPU1 adhesives.

Figure 2a shows the test of the shear strength of plywood bonded with SNIPU1 adhesives prepared with different proportions of PEI-10000. As the amount of PEI-10000 increases, the shear strength of the adhesive improves, with an optimal shear strength observed at a PEI-10000 content of 45% (*w/w* of sucrose). This increase is due to the greater proportion of urethane bonds formed. This, coupled with hydrogen bonding, leads to more six-membered ring structures, thus enhancing bond stability, hydrolytic resistance, and chemical resistance, hence contributing to the improved shear strength. However, as the PEI-10000 content continues to increase, the shear strength decreases. This may be due to the high viscosity of PEI-10000 itself, which leads to higher viscosity in the synthesized adhesive as its content increases. High viscosity also hinders application and inhibits the adhesive’s penetration into the wood surface, this being one of the reasons for the observed decrease in bonding strength, as shown in Figure 2b.

**Figure 3:**
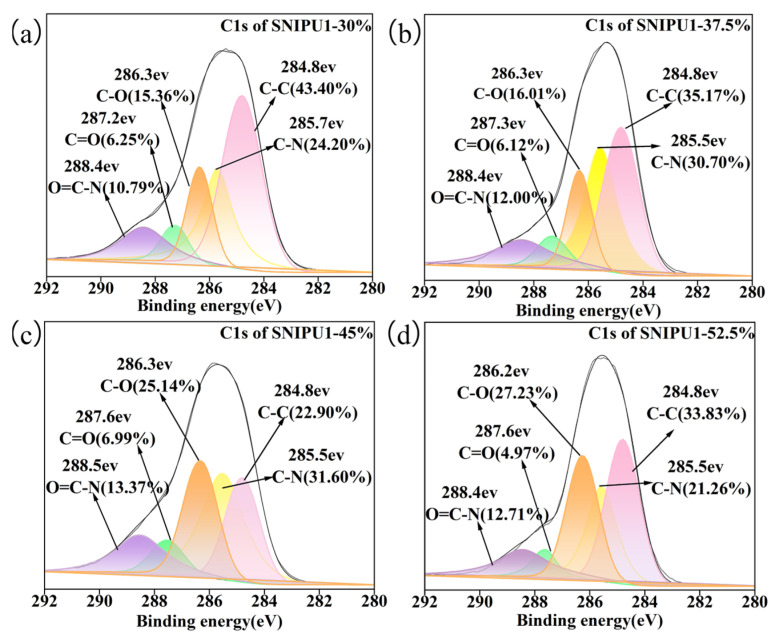
(**a**) C1s XPS spectra of SNIPU1-30% adhesive. (**b**) C1s XPS spectra of SNIPU1-37.5% adhesive. (**c**) C1s XPS spectra of SNIPU1-45% adhesive. (**d**) C1s XPS spectra of SNIPU1-52.5% adhesive.

Figure 2c shows that the FTIR spectra were used to identify the structure and functional groups of the SNIPU1 adhesive. The 1614 cm^−1^ peak is the absorption peak from the C=O stretching vibration. It can be observed that SNIPU1-45% presents a more pronounced absorption peak, which indicates that a greater number of carbamate structures has been generated, with a higher proportion of six-membered rings, and an enhanced overall performance. Figure 3 shows the XPS spectra of adhesives with different PEI-10000 additions, as well as the fraction of distinct chemical bonds in the spectra. It shows that SNIPU1-45% presents the highest O=C-N peak area (13.37%), while the other three adhesives show lower areas: 10.79% for SNIPU1-30%, 12.0% for SNIPU1-37.5%, and 12.71% for SNIPU1-52.5%. This further shows that SNIPU1-45% produces a higher proportion of carbamate structures. Thus, the XPS analysis results confirm the FT-IR spectroscopy data to explain why SNIPU1-45% performs so well. The results of XPS spectral analysis and FT-IR spectral analysis further confirm the successful preparation of the NIPU adhesive.

Figure 2d shows the DSC analysis performed to study the effect of the PEI-10000 content on the thermal properties of the adhesive. Compared to other adhesives, the SNIPU1-45% adhesive exhibits a lower curing onset temperature (45 °C), which indicates a faster curing rate and a greater ability to cross-link more rapidly under the same conditions. Additionally, the SNIPU1-45% adhesive shows a larger integrated area of the DSC curve and a bigger exothermic peak, resulting in a more intense curing exotherm and more complete curing. This contributes to its superior performance. An endothermic peak appears after 200 °C, primarily due to the evaporation of moisture in the adhesive and the decomposition of the adhesive.

### 2.3. Effect of Modifier Type on SNIPU Adhesive at the Lower Hot-Pressing Temperature of 180 °C

**Scheme 1:**
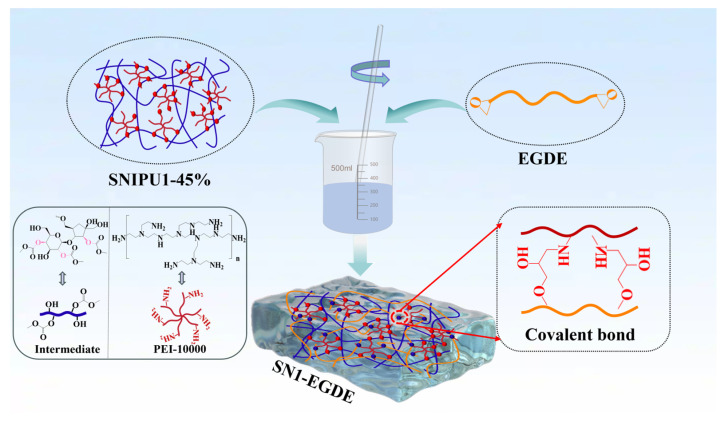
Schematic representation of the preparation of the SN1-EGDE adhesive and the formation reaction of the SN1-EGDE adhesive structure.

**Figure 4:**
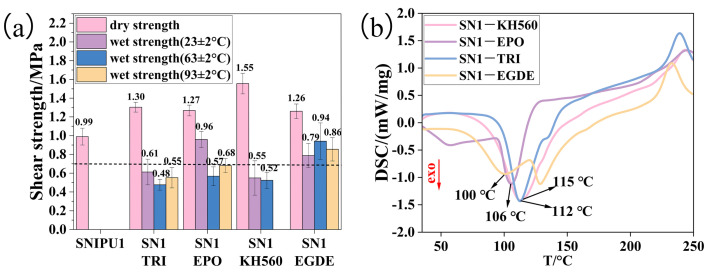
(**a**) Shear strength of SNIPU1 and SN1E adhesive under hot pressing at 180 °C. (**b**) DSC curves of SN1 adhesives.

What described above was carried out at a hot-pressing temperature of 200 °C. Too high a hot-pressing temperature may damage the wood, thus it is crucial to reduce the hot-pressing temperature. To minimize the hot-pressing temperature of the SNIPU adhesive, a further cross-linker has been added to optimize the adhesive performance. The performance of the modified adhesive was then tested at 180 °C. The ternary ring structure of epoxy cross-linkers is easily opened, thus making them extremely reactive and suitable for usage as polymer cross-linkers producing stable covalent bonds [36]. Therefore, in this work, four epoxides, namely EGDE (The reaction scheme is shown in Scheme 1), an epoxy resin, a commercial epoxysilane (KH560), and trimethylolpropane triglycidyl ether were used to modify the SNIPU1 adhesive [37]. Figure 4a shows the effect of adding the same proportion of epoxide on the shear strength of SNIPU1. The plywood prepared with the adhesive modified by EGDE exhibited good wet shear strength at a hot-pressing temperature of 180 °C. In contrast, the unmodified SNIPU1 adhesive and the adhesives modified with the other three types of epoxides showed poorer wet shear strengths.

Figure 4b shows the DSC analysis of the thermal properties of the adhesive after the addition of different epoxides. Under the same curing conditions, the curing temperatures of the SN1-EGDE, SN1-EPO, SN1-TRI, and SN1-KH560 adhesives were 100 °C, 106 °C, 112 °C, and 115 °C, respectively. The SN1-EGDE adhesive exhibited the lowest curing temperature, suggesting its superior performance and effective curing. Therefore, the bonding performance of the SN1-EGDE adhesive is better than that of the other adhesives. An endothermic peak appears after 200 °C, primarily due to the evaporation of moisture in the adhesive and the decomposition of the adhesive.

**Figure 5:**
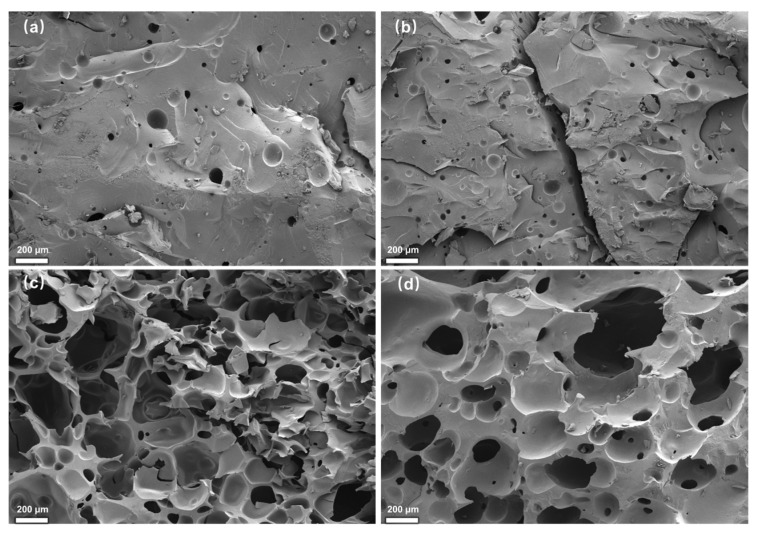
SEM image of SN1-EGDE (**a**), SN1-KH560 (**b**), SN1-TRI (**c**), SN1-EPO (**d**).

Figure 5 shows the SEM images of the SN1 adhesive. It can be clearly observed that SN1-TRI and SN1-EPO (Figure 5c,d) contain numerous pores, which were formed by the volatilization of substances during the high-temperature curing process. These volatiles disrupt the cross-linked network structure of the adhesive, negatively affecting its performance [38]. The SEM image of the SN1-KH560 adhesive (Figure 5b) shows an uneven surface with significant cracks, which may facilitate water penetration and lead to the breakdown of the cross-linked structure, resulting also in a weaker bonding performance. The SEM image of the SN1-EGDE adhesive (Figure 5a), shows noticeably fewer pores and cracks compared to the other three. Its smoother, denser structure effectively prevents water infiltration in humid environments, contributing to its better bonding performance [39]. All this further supports the rationale for selecting EGDE as the additional cross-linking agent.

### 2.4. The Effect of the Amount of the Modifier EGDE on the Adhesive

**Figure 6:**
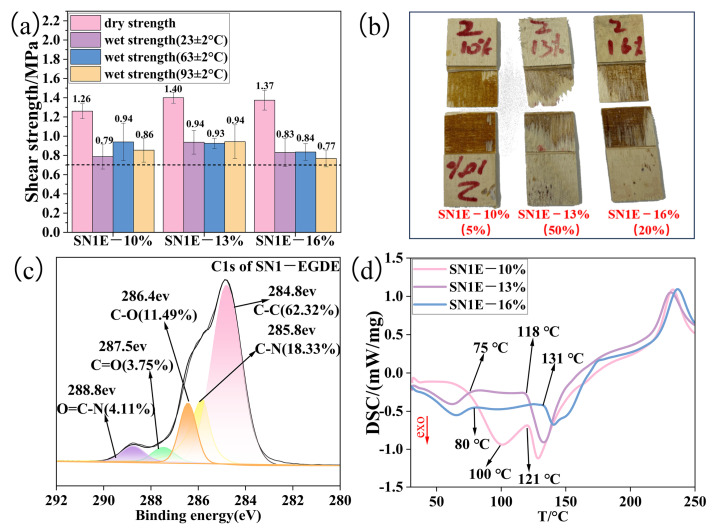
(**a**) Shear strength of SN1E adhesive under hot pressing at 180 °C. (**b**) Plywood dry shear strength test diagram. (**c**) C1s XPS spectra of SN1-EGDE adhesive. (**d**) DSC curves of SN1E adhesives.

Figure 6a shows the shear strength of plywood bonded with adhesives containing different amounts of EGDE when hot pressed at 180 °C. When the EGDE content was between 10% and 16% (*w/w* of SNIPU1), the SN1E-13% adhesive exhibited the best shear strength, with a dry strength of 1.40 MPa and a wet shear strength exceeding 0.90 MPa. This is because EGDE is a low-molecular-weight epoxy compound. Compared to existing methods, the increased addition of EGDE introduces more ether bond structures, enhancing the toughness of the system, thereby reducing the brittleness of the cured adhesive and improving its impact resistance. Additionally, the increase in EGDE reduces intermolecular forces within the adhesive, lowering its viscosity and improving its fluidity, making it easier to apply and penetrate the plywood. A higher EGDE content allows the amino groups in the adhesive to react more fully with the epoxy groups of EGDE, forming a denser cross-linked network. This is the key reason for the significant improvement in adhesive performance under a hot-pressing temperature of 180 °C. However, there are also disadvantages. With a further increase in EGDE content, the wet shear strength of SN1E-16% decreases, possibly due to excessive EGDE reducing the cross-linking density. Moreover, excessive adhesive penetration into the plywood during application results in an overly thin cured adhesive layer, leading to decreased strength. Additionally, an excessive amount of EGDE increases the hydrophilicity of the adhesive, resulting in a higher water absorption rate, which accelerates aging in humid environments [40,41]. Figure 6b shows the physical image of the plywood dry shear strength test, with the wood failure rate indicated in parentheses. The results demonstrate that the sample bonded with SN1E-13% exhibits a higher wood failure rate (50%), indicating superior adhesive performance. Figure 6c shows the XPS spectrum of SN1-EGDE. After the addition of EGDE, the proportion of C-N (18.33%) and O=C-N (4.11%) in SN1-EGDE in the adhesive decreased compared to that before the addition (Figure 3), which may be due to the reaction of the adhesive SNIPU1 with EGDE to introduce more other groups. This confirms the smooth progress of the reaction and validates the reason for the improved strength.

Figure 6d shows the DSC analysis of the thermal properties of adhesives with different amounts of EGDE. SN1E-13% exhibited an exothermic reaction early in the test, which may be due to the reaction between the added EGDE and the adhesive, and the exothermic reaction ended at 75 °C. The larger exothermic peak area observed in the figure indicates a faster and more complete reaction, allowing the adhesive network to form more rapidly and densely. In contrast, the exothermic reaction for SN1E-10% occurred later and did not end until 121 °C, which may be due to the smaller amount of EGDE added, resulting in a smaller reaction contact area. SN1E-13% also began a second exothermic reaction at 118 °C, earlier than SN1E-10% (121 °C) and SN1E-16% (131 °C), indicating that SN1E-13% cures more quickly between the plywood layers. Furthermore, SN1E-13% has a larger exothermic peak, more thorough and complete curing, a stronger exothermic reaction, and a denser bond between the plywood layers, which is one of the reasons for its superior strength. An endothermic peak appears after 200 °C, primarily due to the evaporation of moisture in the adhesive and the decomposition of the adhesive.

## 3. Materials and Methods

### 3.1. Materials

The sucrose was procured from Guangdong Guanghua Technology Co., Ltd. Polyethyleneimine (PEI, Mw = 10,000 Da) was purchased from Adamas Beta (Shanghai) Chemical Reagent Co., Ltd. (Shanghai, China). Dimethyl carbonate (AR, ≥99%), polyethyleneimine (PEI, Mw = 1800 Da), hexanediamine, diethylenetriamine, sodium dodecylbenzene sulfonate, ethylene glycol diglycidyl ether (EGDE), trimethylolpropane triglycidyl ether, and epoxy resin were all purchased from Shanghai Macklin Biochemical Technology Co., Ltd. (Shanghai, China). The silane coupling agent KH560 was purchased from Shanghai Yuanye Biotechnology Co., Ltd. (Shanghai, China).

### 3.2. Preparation of SNIPU Adhesives

A total of 40 g of sucrose, 30 g of water, 36 g of dimethyl carbonate and 1.2 g of sodium dodecylbenzene sulfonate were added into a flask and heated up to 70 °C to keep warm and react for 2 h, then the polyamine compound was added and heated up to 90 °C, and the temperature was timed to react for 30 min to obtain the sample. In this study, four polyamine compounds, PEI-10000, hexanediamine, diethylenetriamine (DETA), and PEI-1800, were used to prepare the adhesives, and the prepared samples were named SNIPU1, SNIPU2, SNIPU3, and SNIPU4, respectively. Upon adjusting the PEI-10000 addition to 30%, 37.5%, 45%, and 52.5% (*w*/*w*, based on the weight of dry sucrose), the resulting adhesives were designated SNIPU1-30%, SNIPU1-37.5%, SNIPU1-45%, and SNIPU1-52.5%, respectively. When fixed at SNIPU1-45%, the samples to which the cross-linker ethylene glycol glycidyl ether, alkyl coupling agent KH560, trimethylolpropane triglycidyl ether, and epoxy resin were added were designated SN1-EGDE, SN1-KH560, SN1-TRI, and SN1-EPO, respectively. Meanwhile, when fixed as SN1-EGDE, the amount of addition was varied to 10%, 13%, and 16% (*w/w*, based on the mass of SNIPU1-45%), and the samples obtained were designated SN1E-10%, SN1E-13%, and SN1E-16%, respectively.

### 3.3. Preparation of Three-Layer Plywood and Bonding Strength Test

The adhesive was uniformly applied to the veneer surface (size 180 mm × 110 mm × 2 mm) at 300 g/m^2^ glue application volume, and the three-layer plywood was subjected to a hot-pressing process at 200 °C and 1.2 MPa pressure for a duration of 6 min. The prepared samples were left at room temperature for 24 h, after which they were cut into pieces measuring 100 mm × 25 mm. They were then tested in accordance with China national standard GB/T 9846-2015 (≥0.7 MPa). Each test was repeated 6 times.

### 3.4. Viscosity Evaluation

Determination of viscosity of SNIPU1 adhesive at room temperature using NDJ digital viscometer (Shanghai Nirun Intelligent Technology Co., Ltd., Shanghai, China). The test was performed using rotor 4, repeated three times at a rotation speed of 6 RPM, and the average value was calculated.

### 3.5. Water Resistance Test of Cured Adhesives

The SNIPU adhesive samples were subjected to a two-hour baking process at 200 °C in an oven. Subsequently, the samples were immersed in water at temperatures of 93 °C and 63 °C for a period of three hours, after which they were dried in an oven at 80 °C for several hours until a stable weight was achieved. The water resistance of the cured adhesives was calculated using the following formula:

(1) $$\mathrm{water}\;\mathrm{resistance}\;\mathrm{of}\;\mathrm{cured}\;\mathrm{adhesives}\;(\%)=\frac{m}{M}\times 100$$

where M and m are, respectively, the mass before and after immersion.

### 3.6. Fourier Transforms Infrared (FT-IR) Spectrosco

The adhesive was dried to a constant weight in an oven at 200 ± 2 °C and then ground into a powder for FTIR analysis. The powder was mixed with KBr powder at a mass ratio of 1:100 and pressed into a sheet-like form using a mold, and analyzed using a Varian 1000 spectrometer (Varian Inc., Palo Alto, CA, USA). The scanning range was 4000–500 cm^−1^, with a resolution of 4 cm^−1^ and 32 scans.

### 3.7. X-Ray Photo Spectroscopy (XPS) Analysis

The XPS analysis was conducted using the Thermo Scientific K-Alpha+ (Thermo Fisher Scientific, Waltham, MA, USA), with a monochromatic Al Kα source having an energy of 1486.6 eV. The pass energy for narrow scan was set at 50 eV, with a step size of 0.100 eV. The binding energy was calibrated using the C1s peak of carbon at 284.8 eV to compensate for charge correction, and the spectra were processed using Thermo Advantage software (5.9921).

### 3.8. Differential Scanning Calorimetry (DSC) Analysis

DSC analysis was conducted using a NETZSCH DSC 204 F1 (Netzsch Group, Selb, Germany) differential scanning calorimeter, with a heating program from 30 °C to 250 °C at a rate of 10 °C/min, in a sealed alumina crucible, while maintaining a constant nitrogen flow atmosphere (20 mL/min).

### 3.9. Scanning Electron Microscopy (SEM)

The dried adhesive samples were cut into rectangles with a flat cross-section, measuring 0.5 cm in length, 0.5 cm in width, and less than 1 cm in height. The surface of the samples was then sprayed with gold and examined under a ZEISS Sigma 300 scanning electron microscope in Germany (ZEISS, Jena, Germany), with an accelerating voltage of 5 kV.

## 4. Conclusions

In the work presented, a novel non-isocyanate biomass sucrose-based adhesive was successfully synthesized by using PEI-10000 (45% sucrose weight) as the primary raw material, with modifications in the type and quantity of polyamine used. The adhesive demonstrated promising performance, with the NIPU1-45% adhesive exhibiting a dry strength of 1.26 MPa, a 24 h cold water (23 °C) wet strength of 0.90 MPa, and 3 h hot water (63 °C and 93 °C) wet strengths of 0.84 MPa and 0.80 MPa, respectively, at a hot-pressing temperature of 200 °C. Furthermore, by incorporating an epoxy-based EGDE-modified adhesive, the hot-pressing temperature was successfully reduced to 180 °C, while still meeting the national standard GB/T 9846-2015 (≥0.7 MPa), which demonstrates its potential for industrial applications in the wood industry. This study not only simplifies the adhesive production process but also opens a new research direction for non-isocyanate biomass adhesives, contributing to the development of more sustainable and environmentally friendly materials. The findings also expand the understanding of the molecular design and performance optimization of non-isocyanate polyurethanes, which are crucial for addressing the current limitations in the adhesive technology used in wood bonding.

## Data Availability

Data will be made available on request.
